# 2012-2025 Roadmap of I.R.Iran’s Disaster Health Management

**DOI:** 10.1371/4f93005fbcb34

**Published:** 2012-07-16

**Authors:** Ali Ardalan, Mohammad Hossein Rajaei, Gholamreza Masoumi, Ali Azin, Vahid Zonoobi, Mohammad Sarvar, khorshid Vaskoei Eshkevari, Elham Ahmadnezhad, Gelareh Jafari

## Abstract

Objective: In line with Iran’s Comprehensive Health Sector Road Map, the National Institute of Health Research at the Tehran University of Medical Sciences developed the 2012-2025 road map of Disaster Health Management (DHM), including goals and objectives, strategies, activities and related prerequisites. This article presents the process and results of this road mapping project. Methods: The project started with an expanded literature review followed by stakeholder analysis to assess level of interest and impact of related organizations to DHM; STEEP.V methodology to define determinants with a potential impact on Iran’s HDM for duration of 2012 to 2025; strength, weakness, opportunity and threat (SWOT) analysis and formulation of goals and objectives, strategies, activities, and prerequisites. Brainstorming, group discussion and interviews with key informants were used for data collection; nominal group technique was used whenever prioritization was necessary, and Delphi panel methodology was applied for consensus development. Results: STEEP.V analysis revealed the most important Social, Technological, Environmental, Economic, Political and Value-based determinants. Iran’s DHM mission and vision were defined respectively as “Mitigation from, preparedness for, response to and recovery from consequences of natural and man-made hazards at the community level as well as to the health facilities and resources of I.R.Iran” and “In 2025, Iran’s DHM will be the most developed system in the region resulting in the least vulnerability, the highest readiness in health facilities and resources, and the highest and most effective contribution of the Iranian community to disaster resilience”, respectively. Sixteen strategies and related activities, along with the necessary prerequisites, were developed. Conclusions: This was the first attempt at comprehensive strategic planning in the field of DHM in Iran. The current framework provides Iran’s health system with a list of strategies and activities to be considered in operational planning and actions. However, a dynamic process of evaluation and revision is required to ensure that Iran’s health system goals are met by 2025. Address for correspondence: Ali Ardalan, No. 78, Italia Ave, Department of Disaster and Emergency Health, National Institute of Health Research, Tehran University of Medical Sciences, Tehran, Iran. Email: aardalan@gmail.com or aardalan@tums.ac.ir Citation: Ardalan A, Rajaei MH, Masoumi G, Azin A, Zonoobi V, Sarvar M, Vaskoei Eshkevari K, Ahmadnezhad E, Jafari G. 2012-2025 Roadmap of I.R.Iran’s Disaster Health Management. PLoS Currents Disasters. 2012 Jul 16

## Introduction

Iran (the Islamic Republic), located in the central Middle East and covering an area of 1,648 million sq km with a population of more than 70 million, is facing many types of natural and man-made disasters. According to the Global Assessment Report on Disaster Reduction (2009), Iran’s natural disasters risk class has been estimated as 8 out of 10 1. During the last four decades, natural hazards have led to more than 109,000 deaths and 53 million persons affected. Earthquake, flood and drought are the most important natural hazards of the country. The Rudbar-Manjil earthquake (1990), Bam earthquake (2003), Golestan flash floods (2000-2005) and cyclone Gonu (2007) were the most deadly or destructive disasters during recent decades 2. In general, hydro-meteorological hazards have shown an increasing trend in terms of occurrence and damage. EM-DAT has recorded 5,402 deaths due to technological hazards from 1900 to 2011 3, and as of 1^st^ January 2011, Iran hosts the 2^nd^ largest group of long standing refugees in the world, with a total of 1,073,366 refugees 4. Clearly, a great deal of attention to disaster management and risk reduction (DMRR) is demanded in Iran.

Along with global initiatives of strategic planning for DMRR such as the “Hyogo Framework for Action” adopted in January 2005 by the United Nations International Strategy for Disaster Reduction (UNISDR) and those implemented by other countries 5, Iran has set up national frameworks and programs in this regard. The country is divided into nine Disaster Management Poles each comprising two to four provinces. Two main organizations are responsible for DMRR: 1) the National Disaster Management Organization (NDMO), under the Ministry of Interior (MOI) and 2) the National Passive Defense Organization (NDPO), under the Supreme Leader. The National Platform for Disaster Risk Reduction was also established under NDMO to follow the Hyogo Framework for Action (HFA) priorities for action. Both NDMO and NPDO have a Health & Medical Services Taskforce (HMST) and Disaster & Emergency Management Center (DEMC) at the Ministry of Health & Medical Education (MOH&ME), which is the leading agency for both taskforces. MOH&ME consists of 49 Universities of Medical Sciences & Health Services (UMSHS) that are responsible for both higher education and health care delivery in designated geographical areas. Each UMSHS has its own Emergency Operations Center (EOC) collaborating with other organizations responding to disasters. In addition to military forces and other ministries, the Iranian Red Crescent Society (IRCS) is the main response organization and is in charge of international humanitarian assistance by law. The medical branch of Basij organization, under the Revolutionary Guard, is another organization with a prominent role in both national and international responses.

Since the Bam earthquake in December 2003, Iran’s health sector has initiated several DMRR programs, including establishment of EOCs, revising emergency response plans and performing regular table top and operational exercises, conducting hazard and vulnerability assessments in hospitals and primary health care facilities, and expanding insurance coverage of health facilities. Integration of DMRR in the well established national network of primary health care is another imperative program that is under pilot. Targeting current and next generation disaster health practitioners and policy makers, Tehran University of Medical Sciences (TUMS) has taken initiatives to establish disaster research and education departments and to develop a Master of Public Health (MPH) with Disaster Concentration, offered since 2006, and a PhD in Disaster & Emergency Health, offered since 2011 6. Despite all of the above-mentioned efforts, Iran’s health system has a long way to go to achieve an appropriate level of readiness and resilience.

To optimize social, technological and economical services and achieve goals identified in “2025 I.R.Iran’s Visions of Development", the country has found strategic planning and framework development as an imperative means of making that vision come true. The most popular instances of development road mapping in Iran are the “National Scientific Roadmap” initiated since 7 March 2011 7, the “1^st^ to 5^th^ Five-Year National Development Program” 8, and the “Comprehensive Health Sector Road Map” 9 by MOH&ME, which has been in action since May 2011.

In line with the Comprehensive Health Sector Road Map, the National Institute of Health Research at the Tehran University of Medical Sciences was assigned to develop the 2012-2025 road map of Disaster Health Management (DHM), including goals and objectives, strategies, activities and prerequisites. This article presents the process and results of this road mapping project.

## Methods

The project was performed from May to September 2011. To achieve the project objectives, we started with an expanded literature review followed by a) stakeholder analysis to assess level of interest and impact of related organizations to DHM, b) social, technological, environmental, economic, political and value-based (STEEP.V) analysis to define determinants with a potential impact on Iran’s DHM for the duration of the period of 2012 to 2025, c) SWOT analysis to assess strengths, weaknesses, opportunities and threats that favor or disrupt Iran’s DHM according to the most important determinants, d) formulation of goals and objectives, strategies, activities and prerequisites. Brainstorming, group discussions and interviews with key informants were used for data collection; nominal group technique (NGT) was used whenever prioritization was necessary and Delphi panel methodology was applied for consensus development. Whenever scoring was required, average score was considered as final score and rounds of Delphi panel consultations through meetings or e-mail were continued until minimum variance of scores was achieved. An advisory panel was held after each step to review the results and decide on possible disagreements. In total, 47 people from 27 governmental and non-governmental organizations and academia participated in the project process.

For the purpose of common understanding and dialogue throughout the project implementation, “UNISDR Terminology on Disaster Risk Reduction” was applied 10. DHM was also defined as “A systematic process of using administrative decisions, organization, operational skills and capacities to meet the challenge of planning for, responding to, recovering from and mitigating health consequences of disasters”. Emphasizing a proactive approach of Iran’s DHM to disaster risk reduction, the scope of the roadmap was defined to extend beyond health facilities and resources so that reducing risk of disaster at the community level was emphasized as well.

To access and review national and international documents related to study objectives, we used the following search strategy for the period of January 2000 to May 2011. The search for international documents targeted both developed and developing countries with a good reputation of disaster planning, including the United States, United Kingdom, Australia and Canada. Regional countries Turkey, Saudi Arabia and Pakistan were considered in addition. Google and Google Scholar were the search engines used, and PubMed, ISI Web of Science and Scopus were the databases searched. The following key-word combinations were used: “Disaster” OR “Emergency” AND (“Management” OR, “Preparedness” OR “Response” OR “Recovery” OR “Mitigation”) AND (“Framework” OR “Roadmap” OR “Strategic Plan”) AND/OR (“Health” OR Public Health” OR “Health System” OR “Health Sector”). “Disaster Risk Reduction” and “Disaster Resilience” were other key words that were searched in combination with other terms. Before each session, the participants were provided with the results of the literature review to assist the brainstorming process.

Stakeholder analysis was started through listing all organizations that potentially have an interest in and/or impact on Iran’s HDM, followed by rating levels of interest (from 1 to 5) and of impact (from 1 to 3), with higher score revealing higher level of interest or impact.

STEEP.V methodology was initiated by participants’ brainstorming on determinants that might affect Iran’s DHM future in either a favorable or unfavorable manner. A “determinant of DHM” was defined as a factor that could have an impact on elements of disaster risk to health facilities and resources or to the general population, including hazard (exposure, intensity, etc), vulnerability (structural and non-structural) and capacity. Participants were asked to briefly explain the effects and trends of each of the listed determinants. Afterward the participants were guided to group the determinants into six categories: Social, technological, environmental, economic, political and value-based. Delphi technique was applied to predict the strength (from 1 as minimum to 5 as maximum) and direction (by positive or negative sign) of the trend of each determinant until 2025 based on previous experience and current and future situation.

SWOT analysis was performed for each DHM determinant, starting with listing the potential strengths and weaknesses as internal factors, and opportunities and threats as external factors. To determine the health system’s situation for managing SWOT factors, the steps described below were taken. 1) Each participant was provided with two forms, one for internal factors and the other for external factors. 2) On each form, a scoring system from 1 (lowest) to 4 (highest) was applied to rate levels of impact and accountability. “Impact level” was defined as potential impact of each factor on the health system’s functioning according to related determinant; “accountability level” was defined as the health system’s ability to overcome or control the weaknesses/threats or to rely on strengths and/or use opportunities. 3) For any specific factor, the “impact coefficient” = impact level of specific factor / sum of impact levels of all factors. 4) For any specific factor, “final score” = accountability level ´ impact coefficient. 5) The sum of factor final scores were calculated for each participant and for each factor, the sum of final scores of all participants was calculated. Results of SWOT analysis provided a basis for development of goals, objectives, strategies, activities, as well as the legal and administrative prerequisites. Again, brainstorming, Delphi panel methodology and NGT were applied for these purposes.

## Results

Stakeholder analysis of Iran’s DHM (Table 1) revealed the following organizations, centers or offices at the MOH&ME as internal stakeholders with the highest interest and impact: Office of the Deputy Minister for Curative Affairs, Office of the Deputy Minister for Public Health, HMSTs at the NDMO and NPDO, DEMC of MOH&ME, DMRR Unit at the Office of the Deputy Minister for Public Health, Center for Management of Communicable Diseases, Center for Environmental & Occupational Health, and National Institute of Health Research. In addition, NDMO, NPDO, IRCS, and medical branch of Basij organization were recognized as the external stakeholders with the highest interest and impact. Positive interaction, participation, advocacy, information sharing and improvement of coordination mechanisms were found as means for enhancement of stakeholders’ participation and collaboration.

Table 2 presents the most important determinants revealed by STEEP.V analysis that would affect Iran’s DHM planning and functionality until 2025 (numbers in parentheses show the strength of trends and related signs show trend direction).

Iran’s DHM mission and vision were defined, respectively, as “Mitigation from, preparedness for, response to and recovery from consequences of natural and man-made hazards at the community level and to the health facilities and resources of I.R.Iran,” and, “In 2025, Iran’s DHM will be the most developed system in the region demonstrating the least vulnerability, the highest readiness in health facilities and resources, and the highest and most effective contribution to disaster resilience by the Iranian community.” A list of indicators was developed to monitor DHM progress toward this mission (Table 3). In line with the above-mentioned mission and vision, and results of SWOT analysis (provided in full text of the project report) 11, the following strategies and priority programs were developed. In the project report, a list of prerequisites are listed for each strategy to facilitate or support the activities.

**Strategy 1:** Inter-sectoral, all-hazard, whole-health approach in DHM.

*Priority programs*: Development and implementation of DHM framework with an inter-sectoral, all-hazard, whole-health approach; continuation of related training and national conferences on an annual basis with contribution of other sectors

**Strategy 2: **Integration of DHM to primary health care (PHC) network from local to national levels

*Priority program*: Development and ratification of the integration program

**Strategy 3: **Consider passive defense principles in all DHM policy making and planning

*Priority programs*: Expand health system infrastructures and resources based on passive defense principles

**Strategy 4: **Proactive approach of health system to disaster risk reduction considering both intensive and extensive risks with a people-centered orientation

*Priority programs*: Community risk assessment with a focus on organizational vulnerability and capacity; profiling health disaster risks produced by other sectors such as construction, industry, hydrometeorology, and informing the related policy makers and administrators; action plan for proactive approach of health system to disaster risk reduction; active contribution in NDMO’s specialized taskforces in addition to HMST; enhancement of early warning system in health system; enhancement of disaster risk perception throughout the community and other sectors; including a community-based disaster risk management module in the integration program of DHM to the PHC network; enhancement of NGOs with a DHM orientation

**Strategy 5: **Build a comprehensive, balanced and dynamic organizational structure inside MOH&ME so that it is able to use full-scale capacities of all internal and external stakeholders

*Priority programs*: Modify and ratify DHM organizational chart in MOH&ME; conduct annual evaluation of HMST; strengthen specialized committees of HMST through enhancement of participatory mechanisms

**Strategy 6: **Maintain a high standard of structural and non-structural safety in construction and retrofitting****health facilities from health houses to referral hospitals so that they are resistant to the chief destructive hazards

*Priority programs*: Conduct periodic vulnerability assessment of health facilities; reconstruct or retrofit vulnerable facilities; standardize non-structural components of health facilities with respect to disaster resilience

**Strategy 7: **Conduct regular disaster exercises, based on dynamic readiness assessment of health system

*Priority program*: Design, control and evaluate regular disaster exercises based on bi-annual plans from the local to the district, provincial and national levels

**Strategy 8: **Develop an effective coordination mechanism in the prevention, preparedness and recovery phases and an effective command system in the response phase

*Priority programs*: Activate specialized committees of HMST; implement training programs with a focus on teamwork at the community and organizational levels; follow up development of Incident Command System (ICS) from the Higher Coordination Committee of NDMO; strengthen and expand EOCs at the national, polar, provincial and district levels

**Strategy 9: **Improve Disaster Information System (DIS) for both response and developmental phases

*Priority programs*: Develop necessary databases using geographic information systems (GIS) with clear levels of access authorization; conduct surveillance of occurrence and impact of hazards; develop and ratify health system access protocol to the internet bandwidth of other organizations at the time of disasters; develop linkage protocol of other organizations’ databases to MOH&ME’s DIS; follow up development of a multi-layer disaster information system based in NDMO

**Strategy 10:** Expand a multi-layer communication system from field to national level

*Priority programs*: Follow up with the National Radio Communication Organization to provide MOH&ME with a UHF frequency; establish a digital communication system in MOH&ME from field to national level; establish a global positioning system (GPS) in the national Emergency Medical Service (EMS); establish satellite communication system in EOCs; situate a Disaster News Studio in MOH&ME

**Strategy 11: **Establish Disaster Rapid Assessment Teams (DRAT) and Disaster Rapid Response Teams (DRRT) with self-sufficient logistic and financial support

*Priority programs*: Develop DRAT and DRRT standards and protocols including medical and public health teams; develop disaster logistic support structure and process; provision disaster funds and petty cash; establish disaster warehouses in organizational chart of MOH&ME; formulate immediate access protocol to regular health system warehouses at the time of disasters

**Strategy 12: **Develop human resources through recruitment and training of an adequate number of****knowledgeable, skilled and passionate disaster responders

*Priority programs*: Develop appropriate positions within MOH&ME with clear job descriptions; develop and carry on quality training programs to train disaster technicians, officers and nurses (bachelor and master levels); expand existing disaster MPH and PhD programs; develop a training matrix for MOH&ME’s disaster human resources; include disaster training as part of medical, paramedic and public health students’ education; develop standards and protocol of disaster staff recruitment in terms of technical and personal eligibility; activate Chapter 3 of NDMO law regarding disaster responders’ overpayment; complete insurance coverage of disaster responders; motivate human resources by providing recreational facilities, building peer network, and expanding access to updated knowledge and skills; contribute in regional and international training and operational programs and experiences

**Strategy 13: **Evidence-based decision making

*Priority programs*: Develop DHM research network; establish research centers; develop DHM’s research puzzle based on priority needs; develop utilization process of DHM’s research findings and training of both researchers and decision-makers in this regard; support scholar publication and establish peer-reviewed journals

**Strategy 14: **Enhance learning capacity from lessons identified, domestic exercises, and international knowledge and experience

*Priority program*s: Document, analyze and present national and international experiences; debrief experienced human resources leaving MOH&ME; develop best practice models of DHM; develop continuous quality improvement of DHM processes using lessons learned

**Strategy 15: **Expand regional and international relations in terms of education, research, consultancy and operational programs

*Priority programs*: Develop a regional network of educators, researchers and professionals; develop a strategic plan for regional and international relations; develop and carry on training programs focusing on Farsi-speaking countries; develop and carry on training programs in English; enhance distance education facilities; organize regional and international conferences and seminars; operationalize the G5 member states’ work plan, including Afghanistan, Iran, Iraq, Pakistan and WHO; develop a joint virtual academic department including regional countries; modify the national law specifying IRCS’ exclusive role in contributing to international humanitarian assistance efforts so that MOH&ME can contribute as well

**Strategy 16: **Strategic and operational planning along with disaster metrics and evaluation

*Priority programs*: Develop HDM 3- to 5-year strategic plans followed by annual operational plans up to 2025; train policy makers, managers and technical staff for DHM strategic and operational planning; standardize input, process, results chain and related indicators of DHM regarding the current roadmap

## Discussion

This was the first attempt at comprehensive strategic planning in the field of DHM in Iran. In this project we developed a framework for 2012-2025 DHM planning and action. The project benefited from analysis of the main determinants with potential impact on DHM during the next 13 years. However, it was limited by the shortage of supportive local or national evidence in terms of research-driven information, so we had to rely mostly on expert opinions. Nevertheless, by using experts from a wide diversity of disciplines and organizations, we believe that we were able to overcome this limitation to some extent, although it is recommended that more valid information be obtained in order to modify the framework over time.

In line with achieving the goals of the 5^th^ National Development Program, MOH&ME has taken the initiative to improve health system management through a consolidated step by step approach to operational planning. The current framework provides MOH&ME with a list of clear strategies and priority programs to be considered in operational planning and actions.

The well-established PHC network in the country 12 provides DHM with a unique opportunity to develop faster than neighboring countries. The country is also experiencing a health reform focusing on the Family Physician program, which is planned to provide services beyond medical care only. A Family Physician program is actually a team consists of 16 public health members headed by a physician. This can be considered a strength to DHM’s objectives of achieving disaster resilience at the grass roots level.

DHM is, however, dependent on policies and structure of disaster management at the national level, specifically NDMO and NPDO. It is expected that these organizations will support the sectoral programs, including health, based on existing or developing national frameworks and on command and coordination tools and mechanisms.

Timely and effective health response operation is limited by the lack of a well-established emergency logistical aid fund. Although the country proved that it can mobilize resources at the time of disasters, the efficiency of operation remains an imperative issue. At the national level, UMSHS and the Office of the Deputy Minister for Management Development & Resources of MOH&ME have been assigned to provide the logistic support for disaster operations, while DEMC is supposed to have logistic facilities in hand.

The health system has had several successful experiences of response to major disasters, including the Bam (2003) and Lorestan (2006) earthquakes, Golestan floods (2000-2005) and Gonu cyclone (2007) 2. But there are questions about health system readiness by hazard and damage scenarios. MOH&ME has recently initiated two programs concerning risk assessments of hospitals and primary health care facilities that have brought the hope of providing necessary information about hazards, structural and non-structural vulnerability and functional readiness of health facilities. This information would assist the health system in more efficient and effective resource allocation, policy making and planning.

More attention is generally paid to major disasters, but extensive geophysical and hydro-meteorological disasters are threatening the people as well. The country faces hazards of high frequency but low impact, which, by definition, each kill fewer than 50 people or damage no more than 500 buildings 1 .

The health system is responsible for the preservation, maintenance and promotion of people’s health, so it must be concerned about any direct or indirect health consequences of disasters. The deaths of more than 100,000 Iranian people due to non-resistant buildings, along with flood-related deaths all over the country because of poor land use planning combined with non-effective early warning systems and evacuation plans, call for a proactive approach by the health system to disaster risk reduction, while emphasizing readiness for response operations. This approach is becoming prominent on the agenda in Iran’s health system and some models of best practices have been developed 13.

By law, IRCS is the only organization that is permitted to act in international humanitarian assistance. MOH&ME is hoping to modify the law and be allowed to contribute in regional and international operations.

To strengthen the capacities of human resources, during the last five years Iran’s health system proceeded to develop, with a leading role of Tehran University of Medical Sciences, an MPH program with disaster concentration, a PhD program in disaster and emergency health, and short-term training courses like Disaster Health Management & Risk Reduction and Refugee Health Care 6.

Since the Bam earthquake (2003), several organizations such as the IRCS and the International Institute of Earthquake Engineering & Seismology (IIEES) have worked on public education to enhance community disaster preparedness. The media have also played a prominent role in increasing community awareness. Despite this fact, a study in three disaster-prone provinces showed low household readiness for natural disasters, specifically earthquakes and floods 14. This highlights the necessity of approaches to translate the community knowledge into practice. The same study revealed that health system interventions based on PHC network capabilities can be effective for this purpose. Focusing on community-based approaches also should be emphasized, with a focus on vulnerable groups, especially elders, whose numbers are increasing within the Iranian community. Improving women’s literacy and participation in the community is an asset for Iran. The equity orientation of the I.R.Iran government focusing on the most socioeconomically vulnerable people is an opportunity for disaster risk reduction programs targeting this part of the community in urban and rural areas.

Despite technological limitations, early warning systems (EWS) of hydro-meteorological hazards are getting established and expanded throughout the country. But the health system needs to be linked to the warning process.

Structural safety of health facilities is a concern of MOH&ME. In this regard, MOH&ME has assessed the structural vulnerability of public hospitals and established a steering committee on safety of health facilities for disasters. The Islamic Parliament has also developed a plan to assist MOH&ME with the required financial resources for retrofitting of vulnerable hospitals during a 10-year period. In addition, all public buildings including health facilities are required by law to be covered by insurance.

A situation of war and political instability in regional countries requires Iran’s health system to be prepared for refugees fleeing to the country, as has been the experience during the last several decades with Iran accepting refugees from Iraq and Afghanistan.

The developed road map requires a dynamic process of evaluation and revision to ensure meeting Iran’s health system goals by 2025.

## Conclusions

This was the first attempt at comprehensive strategic planning in the field of DHM in Iran. The current framework provides Iran’s health system with a list of strategies and activities to be considered in operational planning and actions. However, a dynamic process of evaluation and revision is required to ensure that Iran’s health system goals are met by 2025.

## Tables

**Table 1 - List of Iran’s disaster health management stakeholders by level of interest and impact*:** 

**Internal Stakeholders**
**Group 1** • MOH&ME: Office of the Deputy Minister for Public health, Office of the Deputy Minister for Curative Affairs, HMST at the NDMO and NPD • Office of the Deputy Minister for Curative Affairs at the MOH&ME: DEMC of MOH&ME (including EOC, EMS and Office of Hospital Emergency Departments) • Office of the Deputy Minister for Public Health at the MOH&ME: DMRR Unit, Center for Management of Communicable Diseases, Center for Environmental & Occupational Health, National Institute of Health Research **Group 2 ** • Office of the Deputy Minister for at the MOH&ME: Office of Community Nutrition, Office of Mental and Social Health and Addiction, Office of Population, Family and School Health Office, Unit of Non-Communicable Diseases Management • MOH&ME: Office of the Deputy Minister for Management Development & Resources, Budget Office, Security Office, National Reference Laboratory • UMSHS: Office of the vice chancellor for Curative Affairs, Office of the vice chancellor for Public Health, Related mirror MOH&ME centers/offices, Research Centers **Group 3 ** • MOH&ME: Office of Health Education & Promotion, Office of the Deputy Minister for Education, Office of the Deputy Minister for Research & Technology, Pasteur Institute, Office of the Deputy Minister for Students & Culture, National Blood Transfusion Organization
**External Stakeholders**
**Group 1** • NDMO • NPDO • Iranian Red Crescent Society • Medical branch of Baisj organization **Group 2** • World Health Organization • NGOs (other than IRCS) **Group 3** • Social Security Organization • Ministry of Jihad-Agriculture • Physicians Professional Organization • Nursing Professional Organization • President Office, Deputy for Strategic Planning • Social Welfare Organization • Research Centers (non-affiliated to MOH&ME or UMSHS) • Academia (non-affiliated to MOH&ME or UMSHS) • Other UN agencies and international Organizations

Based on a summary index developed by multiplying levels of interest and impact so that Group 1= Score 11-15, Group 2 = Score 6-10 and Group 3 = Score 1-5, MOH&ME: Ministry of Health & Medical Education, UMSHS: University of Medical Sciences & Health Services, NGO: Non-Governmental Organization, UN: United Nations, EMS: Emergency Medical Service, EOC: Emergency Operations Center, NPDO: National Passive Defense Organization, NDMO: National Disaster Management Organization, IRCS: Iranian Red Crescent Society

**Table 2 - Social, technological, environmental, economic, political and value-based determinants with an impact on Iran’s disaster health management until 2025:** 

**Determinant**	Strength and trend direction*
***Social*** **	
Social capital, community participation and contribution of non-governmental organizations (NGOs)	+2
Gender related vulnerability	+2
Age related vulnerability	-2
Population density	+4
Role of media	+4
Public awareness	+4
***Technological***	
Early warning systems	+3
Search and rescue technology and expertise	+3
Information and communication technology	+3
Learning from previous disasters	+2
Emerging technological hazards	+4
Capable human resources	+3
Health care system	+3
***Environmental***	
Climate and ecosystem change	-3
Population health status	+3
Structural and non-structural safety of health facilities	+2
Safe construction at community level	+2
Emerging diseases with epidemic/pandemic potential	-3
***Economic***	
People’s economic status	-2
Insurance coverage mandated by government	+2
Financial resources allocation by government	+2
Unbalanced development and poor urbanization	-3
***Political***	
National disaster management	+2
Disaster management in health sector	+2
International sanctions	-2
Threats related to hard and soft wars	-4
Legislation and enforced implementation of DRR measures	+2
Socio-political stability of regional countries	-4
***Value-based***	
Considering disaster information as a matter of security	-3
Preventive policies in disaster management	+3
Team working and discipline	-2
Fatalism	-2
Equity-orientation	+3

*Numbers show strength of trends and related signs show trend direction.

**Table 3 - Indicators of Iran’s disaster health management progress:** 

**No**	**Indicator** ****	**2025 Expected value** ****	**Current value**
1	Proportion of health facilities & resources with accepted level of structural and non-structural resistance and appropriate land use planning	100%	<30%
3	Proportion of health facilities compromising accepted level of readiness and capacity responding to disasters	100%	<40%
4	Number of DHM higher education and standard professional training programs	5	1
5	Number of standard and quality professional training courses	10	1
6	DHM’s contribution in knowledge production, based on articles published in international peer-reviewed journals	5%	<1%
7	Proportion of quality and capable DHM’s human resources	100%	<30%
8	Proportion of the community disaster resilience programs with health system’s effective contribution	5	<5
9	Number of regional or international programs with Iran’s health system effective contribution including operational, educational, research and consultancy programs	5	1
10	MOH&ME organizational capability for DHM’s policy making and planning	100%	<30%
11	MOH&ME capability for rapid and effective disaster response operation with adequate logistic support	100%	<30%

DHM: Disaster Health Management, MOH&ME: Ministry of Health & Medical Education
